# Managing Cardiac Amyloidosis in a Rural Cardiology Practice: Bridging Gaps in Specialized Evaluation and Disease-Modifying Treatment

**DOI:** 10.7759/cureus.116194

**Published:** 2026-09-13

**Authors:** Arjun Khunger, Ankita Kapoor, Mohammad Karim, Nikhil Agrawal

**Affiliations:** 1 Hospital Medicine, Sacred Heart Medical Center, Springfield, USA; 2 Oncology, University at Buffalo, State University of New York, Buffalo, USA; 3 Medicine and Cardiology, University of Pittsburgh Medical Center Hamot, Hamot, USA; 4 Cardiology, University of Pittsburgh Medical Center Chautauqua, Jamestown, USA

**Keywords:** attr cardiac amyloidosis, doppler echo, genetic panel testing, heart failure with preserved ejection fraction, internal medicine in rural areas, mri cardiac, technetium-99m pyrophosphate scan, transthyretin cardiac amyloidosis

## Abstract

Cardiac amyloidosis is an underdiagnosed condition characterized by the deposition of amyloid fibrils in the myocardium. Its timely diagnosis remains challenging, particularly in a rural cardiology setting where access to advanced imaging, tissue biopsy, and genetic evaluation is often limited. Early initiation of disease-modifying therapy is critical; however, real-world guidance for clinicians practicing outside tertiary centers is sparse. We describe three consecutive patients diagnosed with cardiac amyloidosis in a rural cardiology clinic, each presenting with non-specific heart failure symptoms that masked classic red-flag features of cardiac amyloidosis. Despite resource constraints, all patients were effectively evaluated using a streamlined diagnostic pathway. Disease-modifying therapy was initiated promptly. This case series demonstrates that early recognition and effective management of cardiac amyloidosis can be achieved in rural settings using a practical and reproducible protocol. We present our experience as a real-world framework to help rural clinicians identify and treat amyloidosis at the earliest opportunity with the aim of improving patient outcomes.

## Introduction

Amyloidosis is a rare infiltrative disorder characterized by the extracellular deposition of insoluble, misfolded protein fibrils that accumulate across multiple organs, including the heart. Cardiac amyloidosis roughly affects 1% of the general population and is predominantly caused by three major subtypes: light-chain (AL) amyloidosis (incidence ~12 per million/year), wild-type transthyretin amyloidosis (ATTR) (prevalence ~141 per million/year), and hereditary ATTR (incidence ~0.3 per million/year) [1]. The principal cardiac pathology is characterized by increased ventricular wall thickness and stiffness leading to restrictive cardiomyopathy and diastolic dysfunction, often accompanied by complications such as atrial fibrillation, conduction system abnormalities, and valvular disorders including aortic stenosis [2].

Given that cardiac amyloidosis frequently presents with nonspecific symptoms such as dyspnea, fatigue, and pedal edema, which overlap with other common cardiac disorders, clinicians must maintain vigilance for diagnostic red flag signs suggestive of cardiac amyloidosis [3]. It includes disproportionately elevated N-terminal pro-B-type natriuretic peptide (NT-proBNP), chronically elevated troponin levels, low voltage or pseudoinfarct patterns on electrocardiogram (ECG), increased ventricular wall thickness without chamber dilation, valvular thickening, and reduced longitudinal strain with relative apical sparing on echocardiogram (ECHO) [3]. However, these findings are neither universally present nor specific to cardiac amyloidosis, particularly in the early stages of disease [4]. Studies have shown a median delay of 13 months in the diagnosis of transthyretin (ATTR) cardiac amyloidosis [5]. A meta-analysis found that approximately 11% of patients with heart failure with preserved ejection fraction (HFpEF) have transthyretin amyloidosis; therefore, screening for cardiac amyloidosis is strongly warranted [6].

Underdiagnosis is even more pronounced in rural settings. Limited access to advanced imaging modalities, such as cardiac magnetic resonance imaging (CMR), technetium-99m pyrophosphate (PYP) scintigraphy, and timely hematologic and genetic testing can delay or prevent definitive diagnosis. Referral pathways to tertiary centers may involve long travel distances, fragmented communication, or significant wait times. These barriers contribute to late-stage presentation, when patients are less responsive to disease-modifying therapy. As treatment options, such as tafamidis, acoramidis, and vutrisiran, have become available, the need for early recognition is more critical than ever. Therefore, practical strategies tailored to rural clinical environments are essential to improve timely diagnosis and outcomes. This case report describes the workup and treatment of suspected cardiac amyloidosis in a rural cardiology clinic, with emphasis on a practical, resource-conscious diagnostic pathway.

## Case presentation

Case 1

A 67-year-old male with a history of hypertension and hyperlipidemia presented to our rural cardiology clinic following an abnormal ECHO obtained for dyspnea and bilateral swelling in the lower legs. He denied chest pain, syncope, or palpitations at presentation but reported six months of exertional dyspnea, corresponding to New York Heart Association (NYHA) class III symptoms [7]. The patient reported mild peripheral neuropathy for 10 years. Laboratory results at diagnosis included an NT-proBNP level of 1380 pg/ml, high-sensitivity troponin level of 52.81 ng/l, creatinine level of 1.04 mg/dl, and hemoglobin of 14 g/dl.

ECHO revealed a left ventricular ejection fraction of 35-40% and severe asymmetric left ventricular hypertrophy (Figure 1).

**Figure 1:**
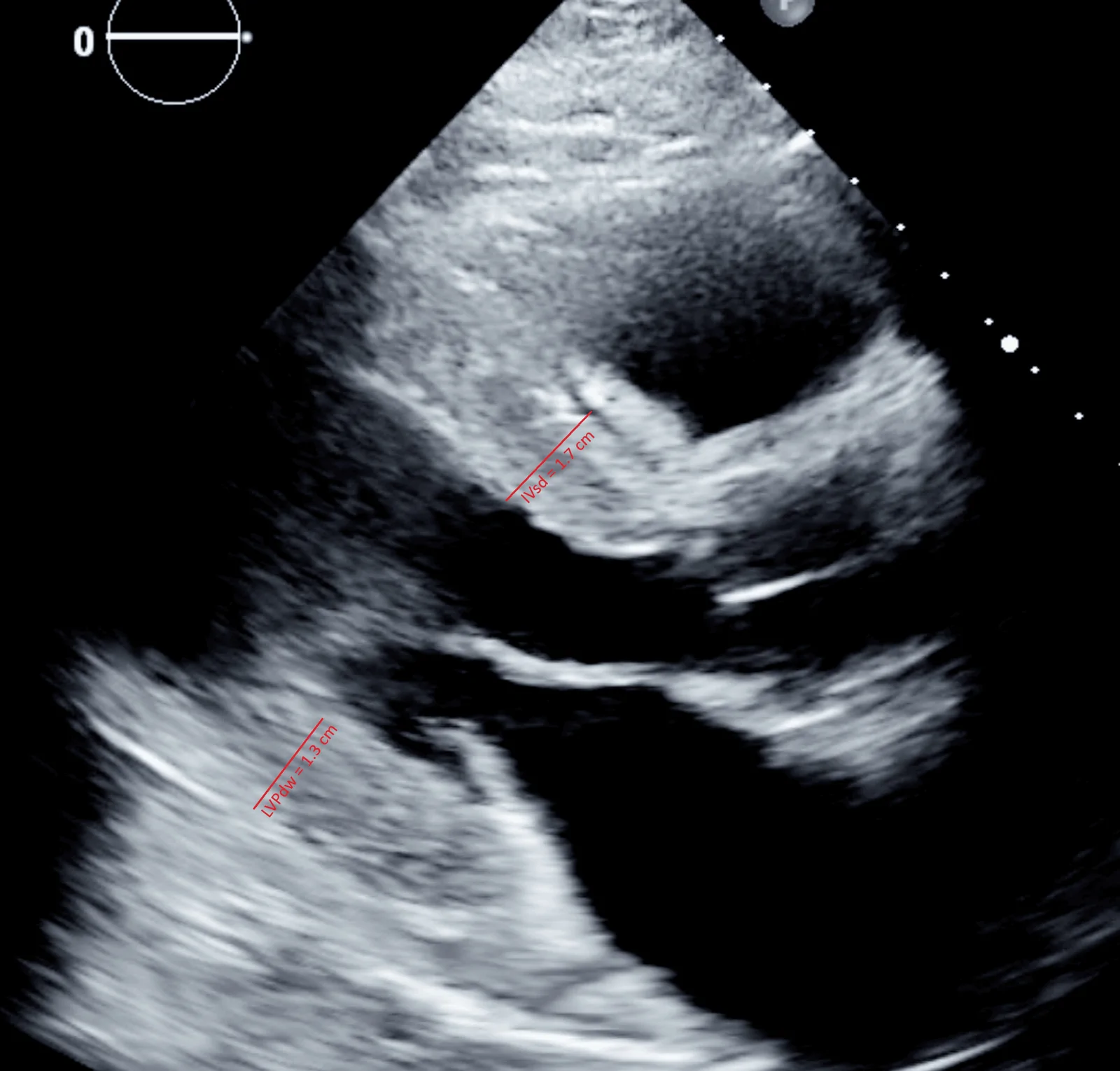
Echocardiogram of patient 1 showing severe asymmetric LV hypertrophy Increased left ventricular (LV) septal thickness noted with a speckling pattern on echo. LVsd (interventricular septal thickness) is 1.7 cm and LVPwd (LV posterior wall diameter) is 1.3 cm. These are labelled in red with red lines.

Additional findings included biatrial enlargement (LAVi is 41 ml/m2 and RAVi is 33 ml/m2), preserved RV function (tricuspid annular plane systolic excursion (TAPSE) is 1.7 cm). Right- and left-heart catheterization demonstrated minimal non-obstructive coronary artery disease, low-normal filling pressures, and mildly reduced cardiac index. 

CMR was done for further evaluation of severe asymmetric left ventricular hypertrophy (IVsd = 1.8 cm and PWd = 1.2 cm), and it demonstrated diffuse subendocardial late gadolinium enhancement of both ventricles, abnormal enhancement of the left atrial wall, and significantly elevated myocardial T1 and extracellular volume (ECV) values. Blood-pool/myocardial inversion on Look-Locker sequences and normal T2 mapping collectively supported an infiltrative process consistent with amyloidosis (Figure 2), with the basal anteroseptal wall measuring 1.8 cm, posterior wall 1.2 cm, and maximal thickness of 1.9 cm at the mid-inferoseptal wall. Wall thickness was notably heterogeneous, with inferolateral myocardial segments measuring as low as 0.7 cm.

**Figure 2:**
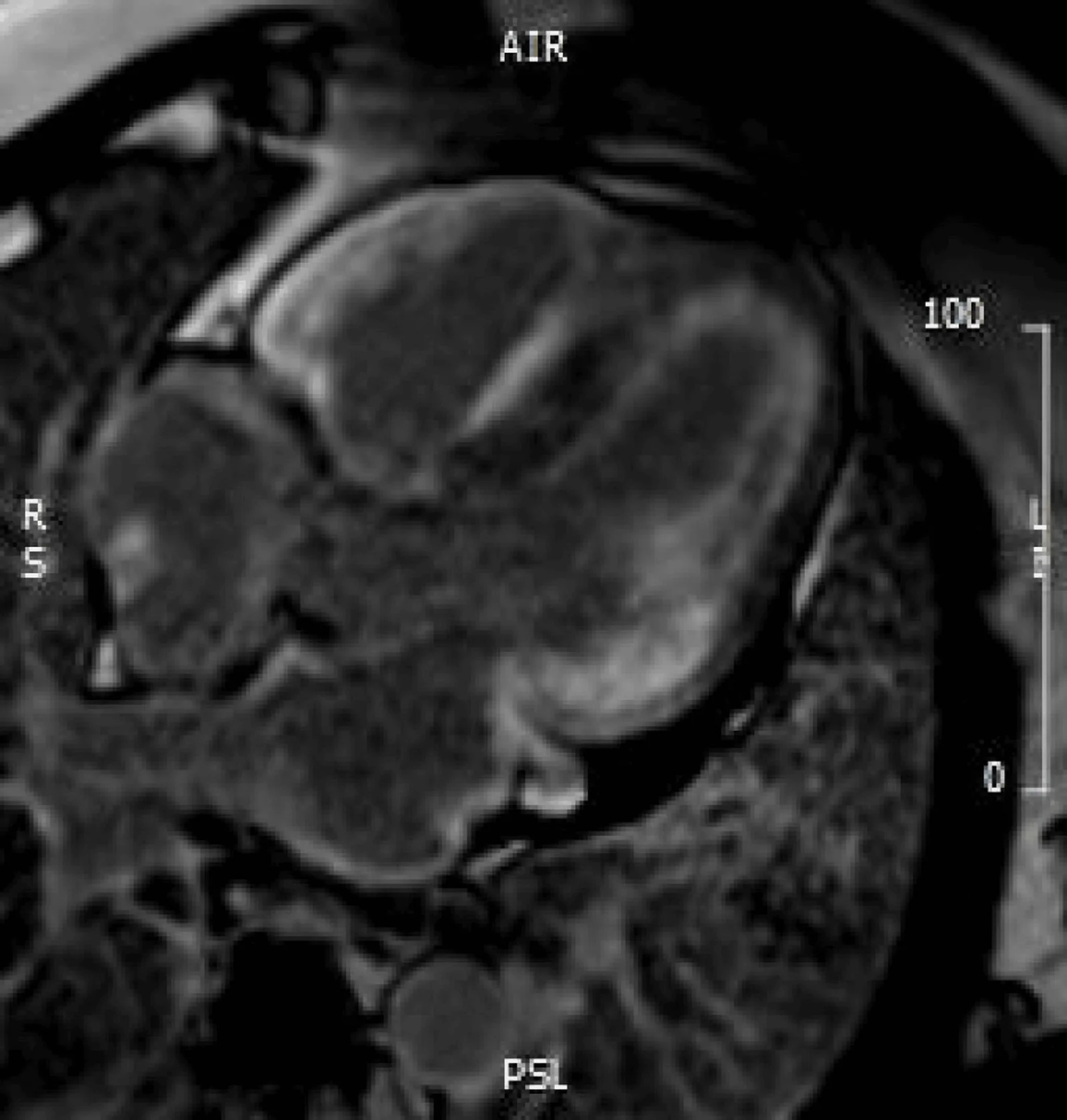
Cardiac MRI (post-contrast Look-Locker) of Patient 1 showing heterogeneous LV wall thickening: mid-inferoseptum 1.9 cm, basal anteroseptum 1.8 cm, posterior wall 1.2 cm, consistent with diffuse myocardial infiltration LV: left ventricular

Technetium-99m pyrophosphate (PYP) scintigraphy showed Perugini grade 3 uptake, with myocardial tracer intensity significantly exceeding rib uptake, strongly suggestive of ATTR cardiac amyloidosis (Figure 3, Figure 4).

**Figure 3:**
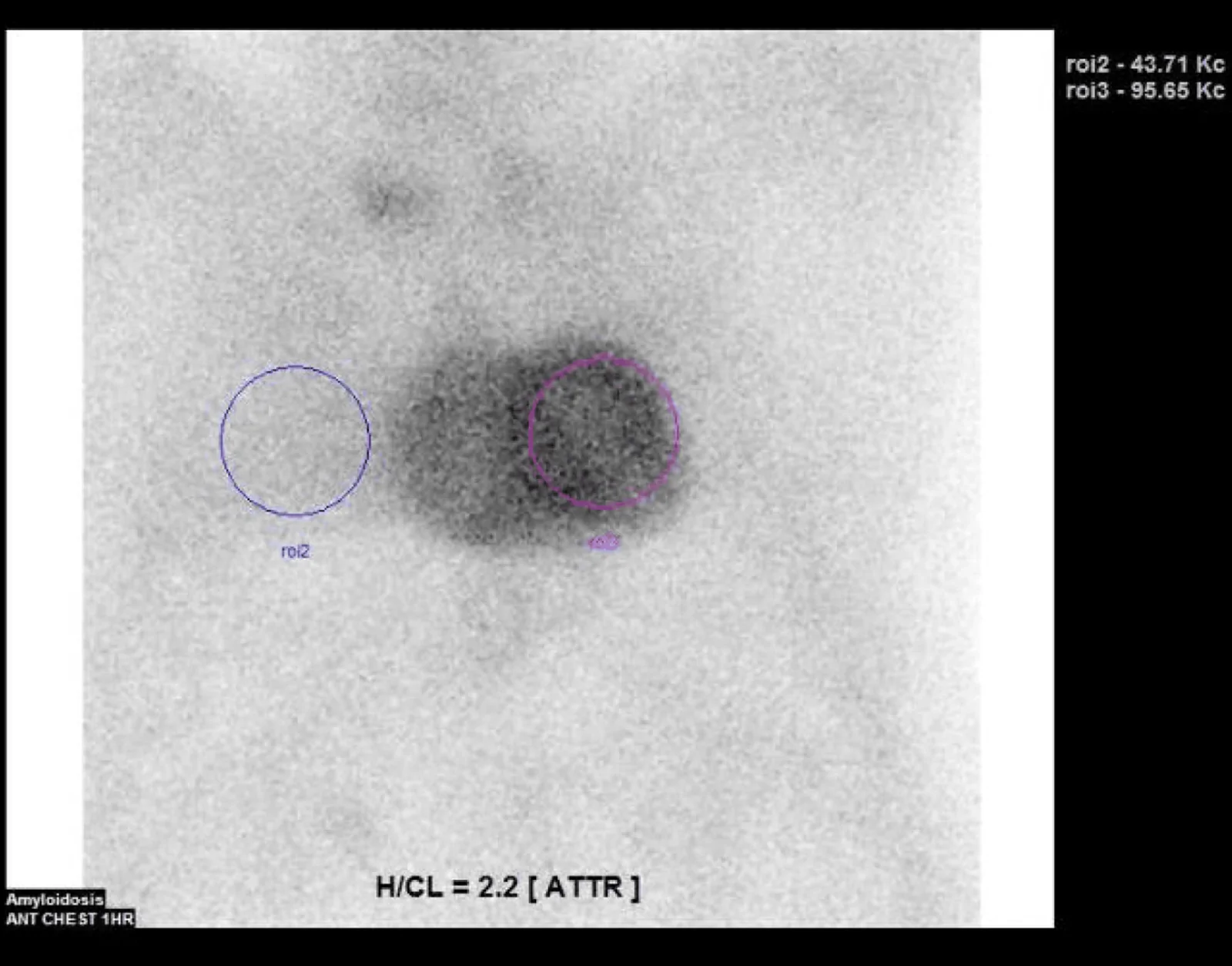
Anterior planar 99mTc-PYP image at 1 h showing increased cardiac uptake with an H/CL ratio of 2.2, consistent with ATTR-CM The dark purple circle on the left, labeled roi2, is the background contralateral lung uptake, versus the left purple circle on the right side, labeled roi3, is the cardiac uptake in the myocardium. PYP: technetium-99m pyrophosphate; H/CL: heart-to-contralateral lung; ATTR-CM: transthyretin amyloidosis cardiomyopathy

**Figure 4:**
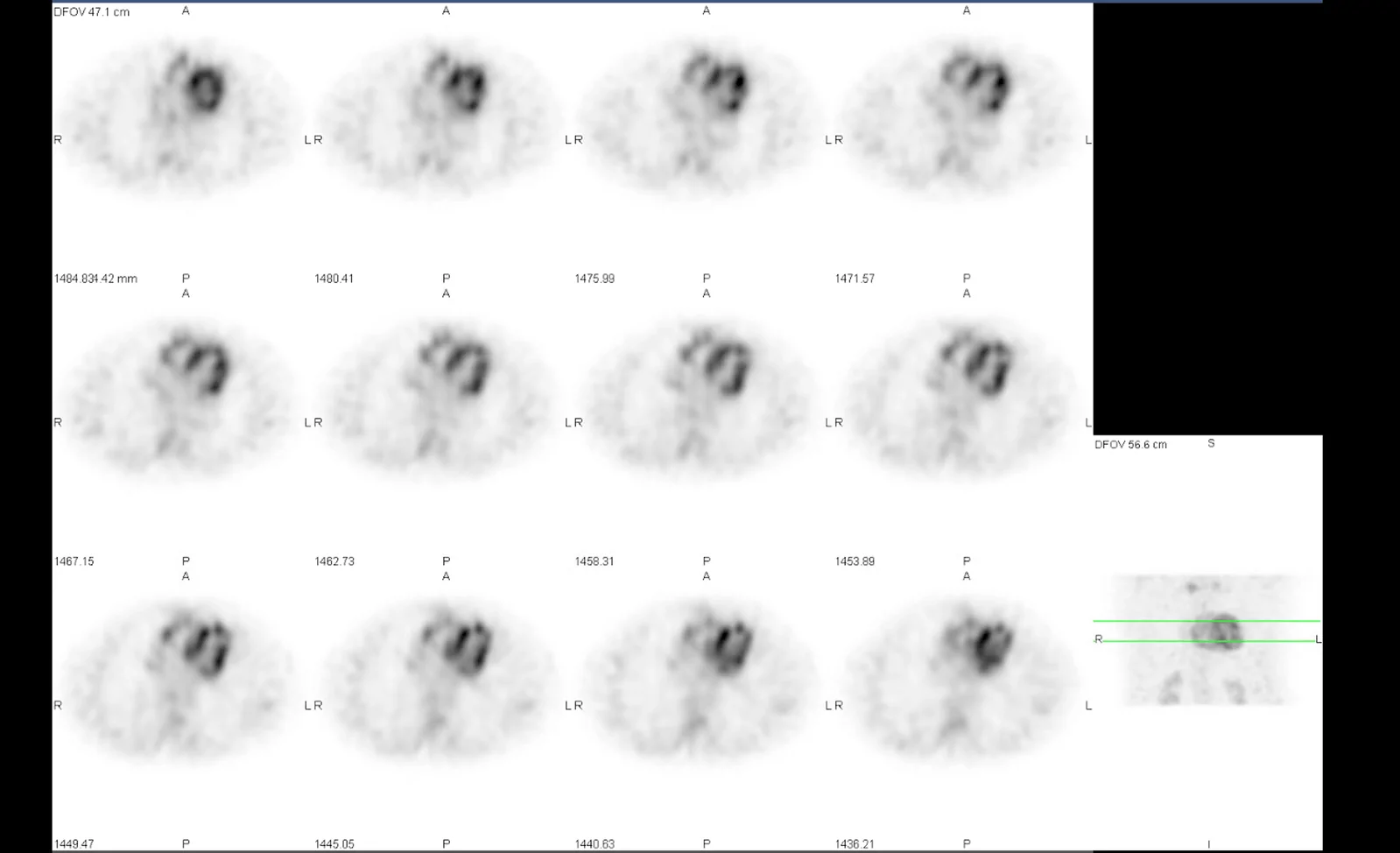
99mTc-PYP cardiac SPECT images demonstrating circumferential radiotracer uptake within the LV myocardium, confirming true myocardial localization and excluding predominant blood pool activity. PYP: technetium-99m pyrophosphate; LV: left ventricular; SPECT: single-photon emission computed tomography

Serum and urine immunofixation, serum protein electrophoresis (SPEP) and urine protein electrophoresis (UPEP) screening, and serum free light chains were all within normal limits, with no laboratory evidence suggestive of a monoclonal protein or underlying AL amyloidosis. Genetic counseling was completed, and transthyretin gene testing returned negative, confirming wild-type ATTR. The baseline 6-minute walk test showed 250 metres with O2 saturation of 96%, and dropped to 92% toward the end of exercise.

The patient was initiated on acoramidis. The patient reported no side effects from therapy. At two months of follow-up, the patient remained clinically stable with a mixed functional trend, no hospitalizations, and continued tolerance of acoramidis. Repeat laboratory values at 2 months showed an improved NT-proBNP level to 1040 pg/ml, high-sensitivity troponin level increased to 62 ng/l, and creatinine remained stable at 1.0 mg/dl. A follow-up 6-minute walk test showed 230 metres compared with 250 m at baseline. Although oxygen saturation improved from 92% at baseline to 95% at the end of exercise, the reduced walking distance indicates a mixed functional trend.

Case 2

An 88-year-old male with a history of severe aortic stenosis requiring transcatheter aortic valve replacement (TAVR), HFpEF, moderate mitral regurgitation, and coronary artery disease managed medically (moderate left anterior descending stenosis without revascularization), presented to our rural cardiology clinic for shortness of breath. He reported progressive bilateral lower-extremity swelling and worsening exertional dyspnea over 12 weeks corresponding to NYHA class II-III symptoms. He denied angina, palpitations, presyncope, or syncope. The patient reported experiencing significant peripheral neuropathy, characterized by bilateral tingling and numbness, persisting over the past 8 years. He did have a history of carpal tunnel syndrome.

ECHO showed preserved LVEF (50-55%) with severe asymmetric LV hypertrophy (LV interventricular septal thickness (LVISd) was 1.7 cm and LV posterior wall diameter (LVPwd) was 1.2 cm), concerning for infiltrative cardiomyopathy (Figure 5).

**Figure 5:**
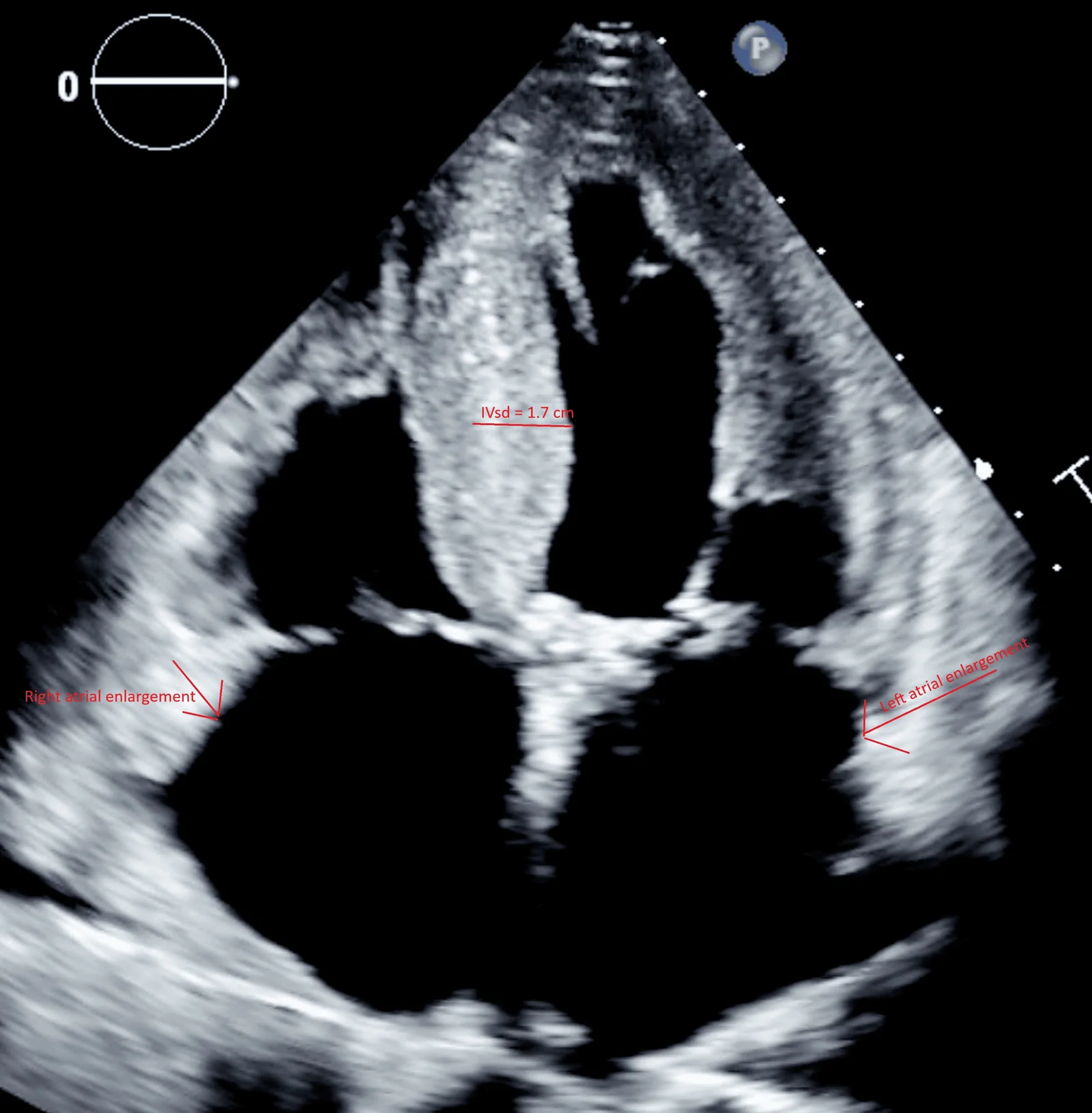
Echocardiogram of patient 2 showing severe hypertrophy of the interventricular septum and severe biatrial dilatation Increased LV interventricular septal thickness (labeled with a red line as IVsd = 1.7 cm) is noted with severe biatrial dilatation (labeled with two red arrows on each side). LV: left ventricular

High-sensitivity troponin T was 39.56 ng/L, and NT-proBNP was elevated at 5289 pg/mL. Renal function remained preserved (creatinine 1.1 mg/dL). Given his progressive symptoms, marked LV hypertrophy with low-voltage EKG, and a history of carpal tunnel syndrome and peripheral neuropathy, a diagnostic evaluation for cardiac amyloidosis was pursued.

A PYP scan demonstrated grade 3 myocardial uptake, confirmed by single-photon emission computed tomography (SPECT), with a heart-to-contralateral ratio of 1.7 at 1 hour, diagnostic for ATTR cardiac amyloidosis (Figure 6 and Figure 7).

**Figure 6:**
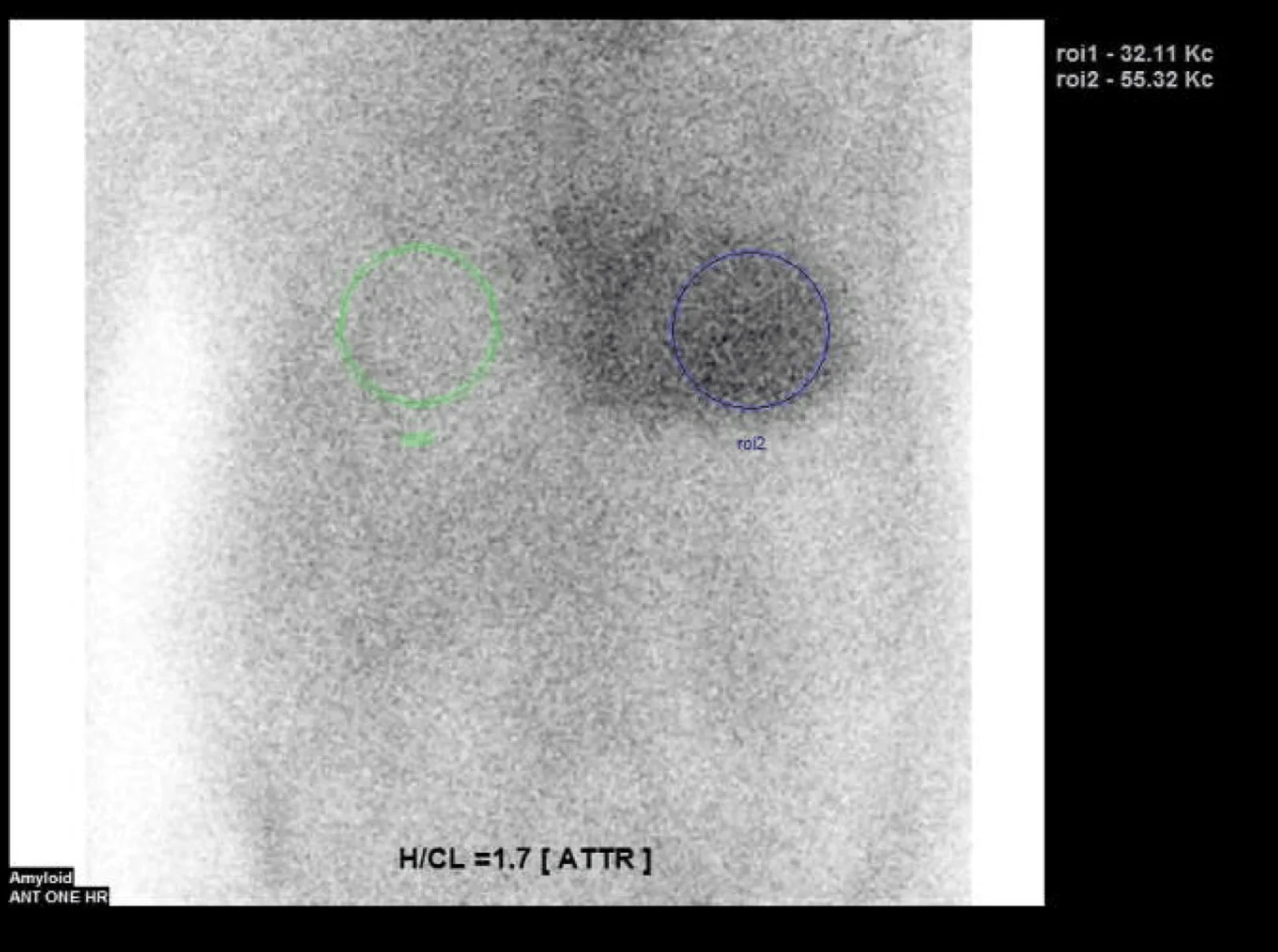
Tc-99m PYP scan showing increased myocardial uptake with a H/CL ratio of 1.7 at 1 hour, consistent with ATTR-CM PYP: technetium-99m pyrophosphate; H/CL: heart-to-contralateral lung; ATTR-CM: transthyretin amyloidosis cardiomyopathy

**Figure 7:**
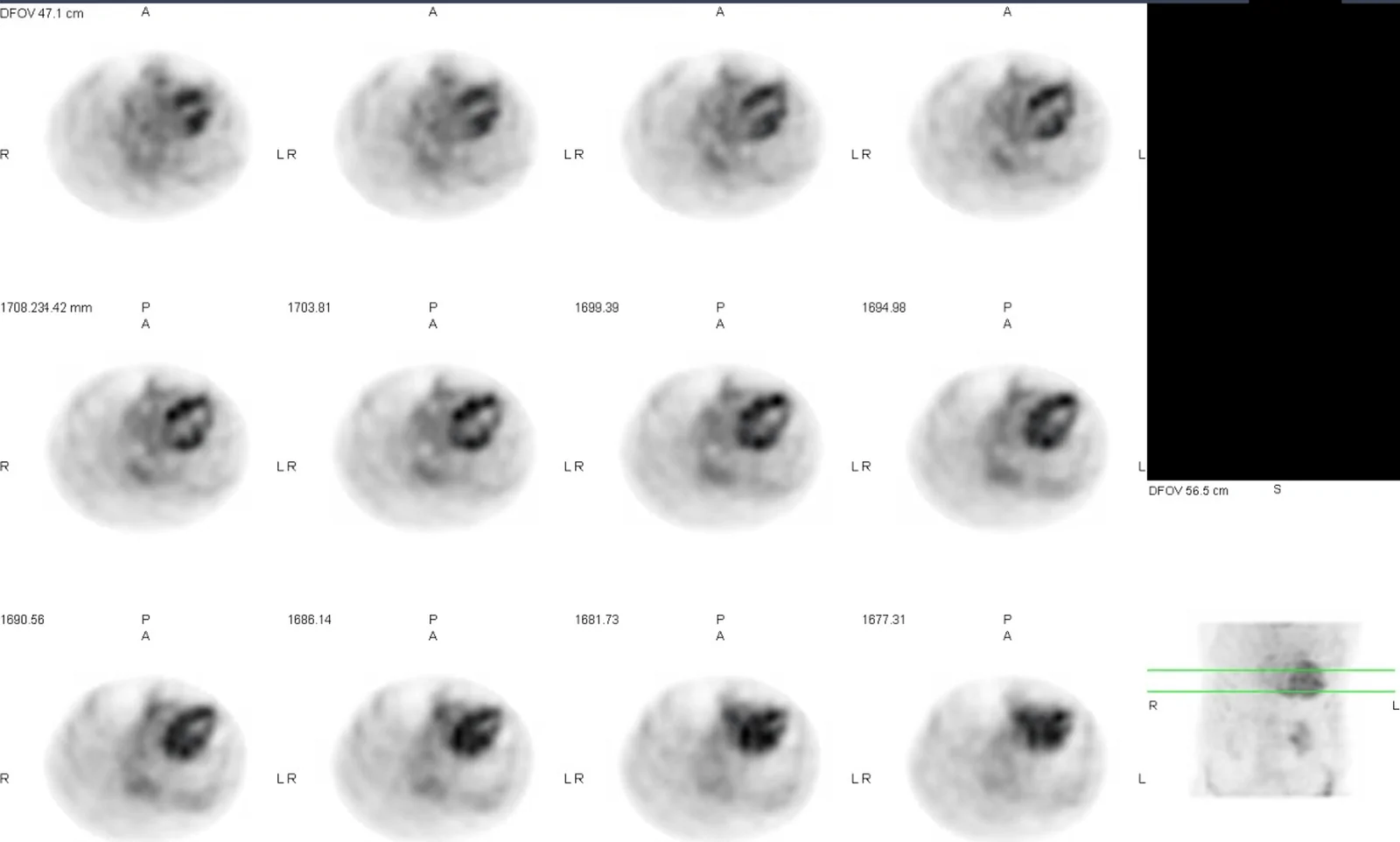
Tc-99m pyrophosphate SPECT imaging demonstrating diffuse LV myocardial radiotracer uptake, consistent with ATTR-CM PYP: technetium-99m pyrophosphate; LV: left ventricular; SPECT: single-photon emission computed tomography

Serum and urine immunofixation electrophoresis revealed no monoclonal protein, and serum free light chains with a kappa/lambda ratio were within normal limits. Collectively, these findings made AL amyloidosis less likely. Genetic counseling and TTR gene testing returned negative, confirming wild-type ATTR. Based on biomarkers and functional status, he met criteria for stage II ATTR cardiomyopathy (Grogan staging system [8]).

The patient was initiated on acoramidis; however, within two weeks, he developed severe gastrointestinal side effects, including refractory diarrhea, profound weakness, and an approximately 10% unintentional weight loss. He was unable to tolerate oral intake and continued to have NYHA II-III symptoms. Given the intolerance to acoramidis and his significant peripheral neuropathy, a transition to vutrisiran was done a few months after obtaining insurance authorization.

New-onset paroxysmal atrial fibrillation with right bundle branch block was noted on ECG on follow-up four weeks later. Given the elevated thromboembolic risk in cardiac amyloidosis, he was started on apixaban (independent of the CHA₂DS₂-VASc score). Rate control was deferred to avoid bradycardia in the setting of restrictive physiology.

At one month of follow-up, the patient remained clinically stable with improved functional capacity, no hospitalizations, and continued tolerance of vutrisiran. Repeat follow-up biomarkers improved. High-sensitivity troponin was 30.2 ng/L, and NT-proBNP was elevated at 1500 pg/mL.

Case 3

An 86-year-old male with a complex cardiovascular history, including two-vessel coronary artery bypass grafting 10 years ago, bioprosthetic aortic valve replacement and mitral valve repair, hypertension, hyperlipidemia, psoriasis, myasthenia gravis, and complete heart block requiring dual-chamber pacemaker 5 years ago, presented to our rural cardiology clinic for evaluation of profound dyspnea and inability to ambulate.

ECHO showed well-functioning bioprosthetic aortic and mitral valves with normal prosthetic gradients and preserved LVEF, but severe LV hypertrophy (LVISD = 2.1 cm and LVPwd = 1.1 cm), raising concern for infiltrative cardiomyopathy. Given progressive HFpEF symptoms and marked LV hypertrophy, a diagnostic workup for cardiac amyloidosis was pursued. ECHO showed a normal ejection fraction of 50- 55% (Figure 8).

**Figure 8:**
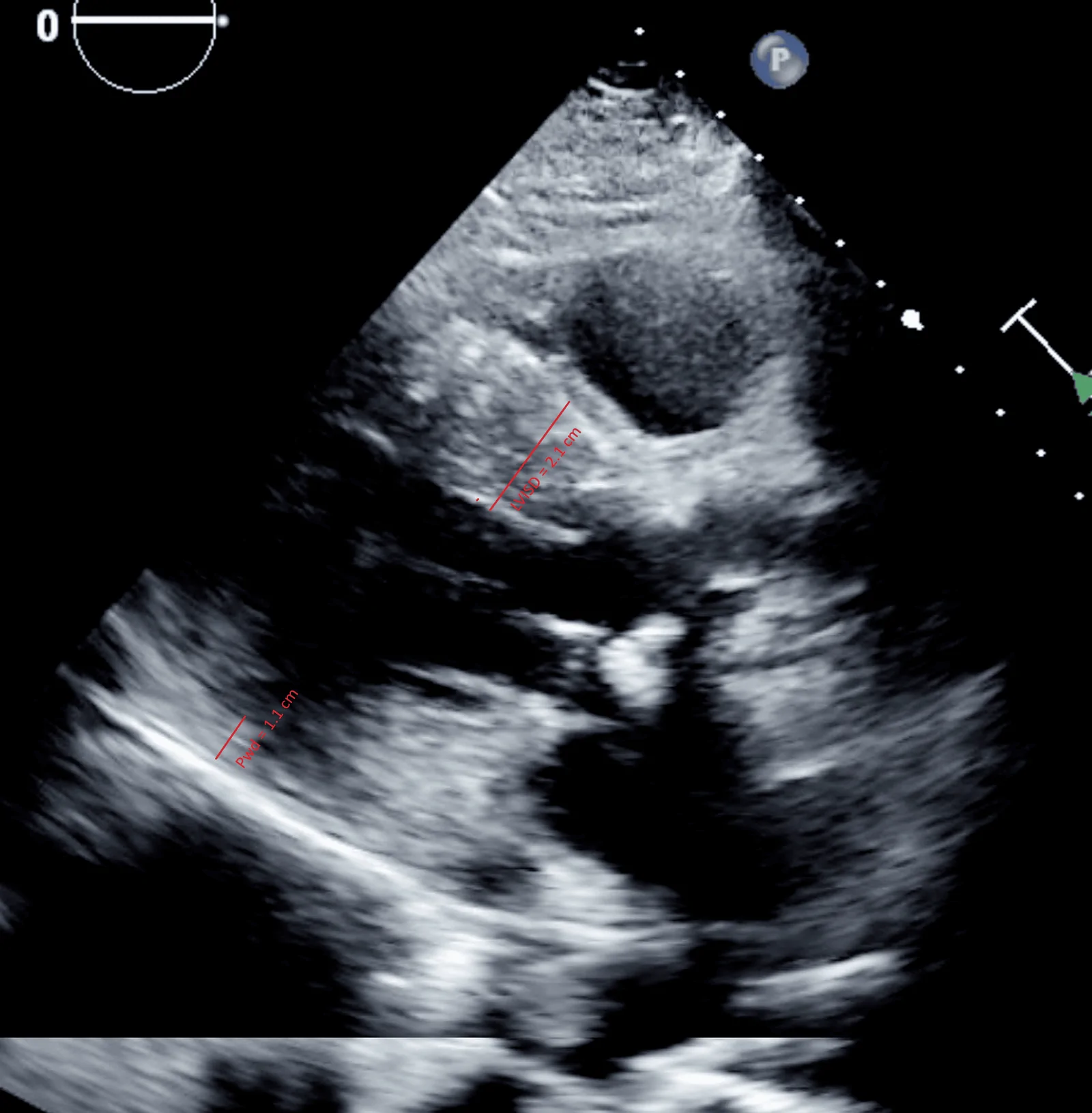
Echocardiogram of patient 3 showing increased LV interventricular septal thickness LVISD = 2.1 cm and LV posterior wall diameter (LVPwd) = 1.1 cm. The red line shows increased LV septal thickness measuring 2.1 cm, noted with a speckling pattern in the interventricular septum. LVISd: LV interventricular septal thickness; LVPwd: LV posterior wall diameter

Cardiac MRI could not be performed due to pacemaker limitations. Serum and urine immunofixation, serum protein electrophoresis (SPEP)/urine protein electrophoresis (UPEP), and serum free light chains were negative, making AL amyloidosis unlikely. Technetium-99m PYP scintigraphy showed grade 3 myocardial uptake, with a heart-to-contralateral (H/CL) ratio of 2.1, confirmed on SPECT to represent true myocardial binding rather than blood pool activity (Figure 9 and Figure 10).

**Figure 9:**
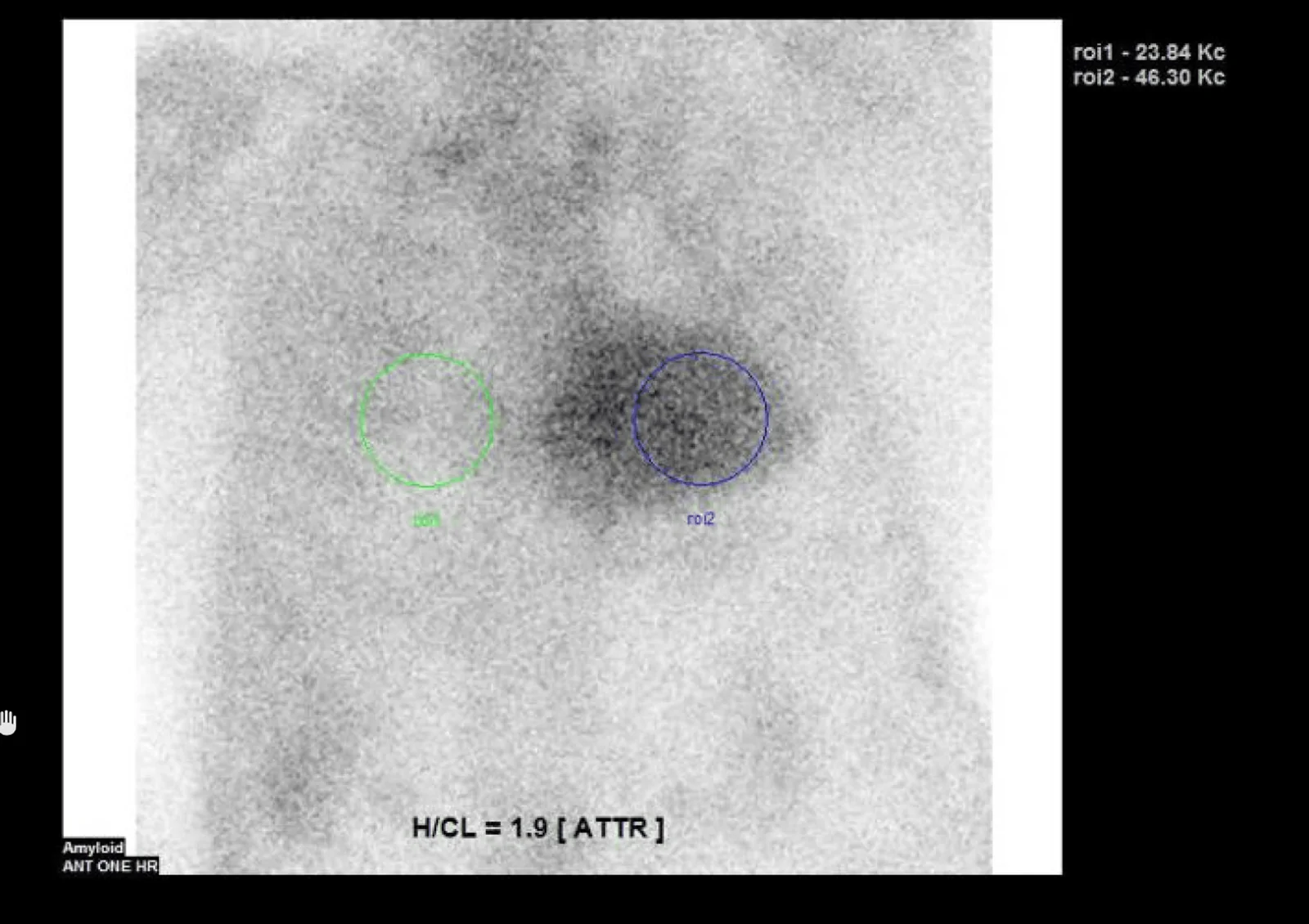
Anterior planar 99mTc-PYP image at 1 h showing increased cardiac uptake with an H/CL ratio of 1.9, consistent with ATTR-CM PYP: technetium-99m pyrophosphate; H/CL: heart-to-contralateral lung; ATTR-CM: transthyretin amyloidosis cardiomyopathy

**Figure 10:**
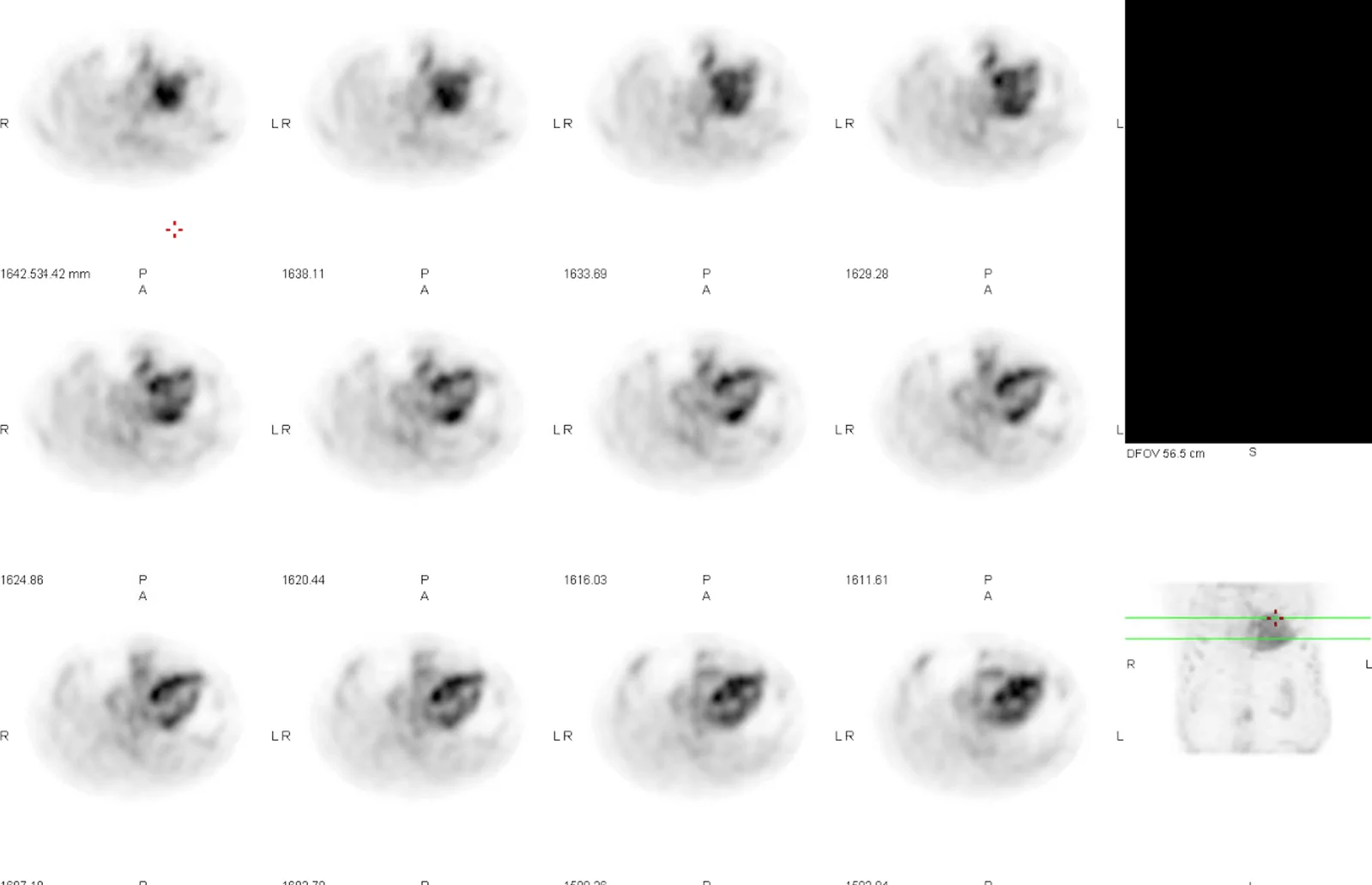
Tc-99m pyrophosphate SPECT imaging demonstrating diffuse LV myocardial radiotracer uptake, greater than background activity, consistent with ATTR-CM PYP: technetium-99m pyrophosphate; LV: left ventricular; SPECT: single-photon emission computed tomography; ATTR-CM: transthyretin amyloidosis cardiomyopathy

Genetic counseling was performed, and TTR gene testing was negative, confirming wild-type ATTR cardiac amyloidosis.

The patient was diagnosed with HFpEF secondary to wild-type ATTR cardiomyopathy. He was initiated on acoramidis, but within three weeks, developed severe gastrointestinal toxicity, which is a known side effect of acoramidis (reported incidence 5-10%). He reported worsening peripheral neuropathy and an exacerbation of myasthenia gravis, requiring inpatient hospitalization and ultimately death from complications of pneumonia. Given his intolerance to TTR stabilizer therapy, he was deemed a candidate for vutrisiran but died prior to the initiation of therapy.

## Discussion

This case series presents a diagnostic pathway and newer therapies employed for patients with ATTR cardiac amyloidosis diagnosed in our clinic (Figure 11).

**Figure 11:**
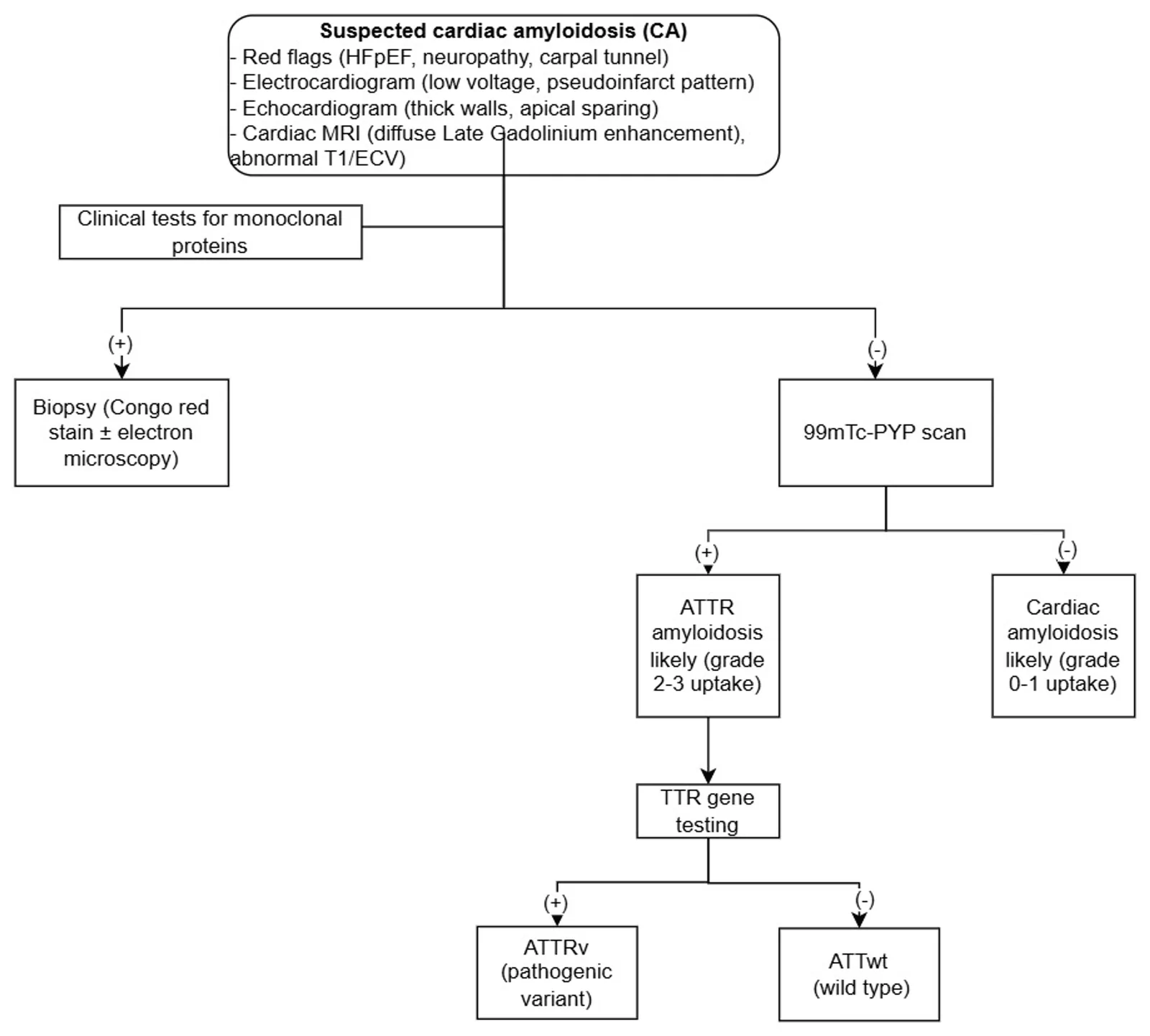
Diagnostic pathway for cardiac amyloidosis 99mTc‐PYP: technetium pyrophosphate; ATTR: transthyretin

Treatment for ATTR cardiomyopathy has rapidly evolved from a single disease-modifying option to a diverse therapeutic landscape that includes high-affinity TTR tetramer stabilizers, RNA silencers, gene-editing agents, and monoclonal antibody-based fibril depleters, with several of these emerging approaches currently under investigation in clinical trials [9]. Tafamidis was the first FDA-approved therapy for ATTR-cardiomyopathy in 2019, emerging from the pivotal ATTR-ACT trial, which showed that early treatment, particularly in patients with milder symptoms, reduced mortality and cardiovascular hospitalizations [10]. In 2024, a next-generation TTR stabilizer, acoramidis, with higher binding affinity and superior reduction in cardiovascular events and improved survival compared with placebo in the ATTRibute-CM trial, was approved by the FDA [11]. In parallel, novel modalities, such as the RNA silencer vutrisiran, were approved by the FDA for patients with ATTR cardiomyopathy in 2025 [12,13].

In our clinic, all patients presented with non-specific heart failure symptoms, such as exertional dyspnea, often attributed initially to common conditions, such as valvular heart disease or ischemic cardiomyopathy, which was eventually ruled out. ECHO revealed severe LV hypertrophy in all cases with apical sparing patterns in some patients. CMR imaging was performed when feasible to enable more detailed evaluation and support the diagnostic evaluation, although CMR is not required to establish the diagnosis of cardiac amyloidosis. 99mTc-PYP scintigraphy was instrumental in confirming the diagnosis, showing strongly positive scans (Perugini Grade 3) in all cases. Finally, AL amyloidosis was considered unlikely in all cases based on negative serum and urine immunofixation and a normal serum free light-chain ratio, recognizing that absolute free light-chain levels may be elevated despite a normal ratio. TTR gene testing was negative in all patients, confirming wild-type ATTR. This highlights the critical need for systematic screening protocols, even in settings where tertiary care access is limited.

Technetium-99m PYP scintigraphy is now a central noninvasive modality in the diagnostic evaluation of cardiac amyloidosis -particularly ATTR cardiac amyloidosis because of its broad availability, relatively low cost, and strong diagnostic performance. As a bone-avid tracer deployable on standard nuclear medicine platforms, it can be implemented in most centers without specialized infrastructure. Furthermore, semi-quantitative measures, such as the H/CL ratio, target-to-background ratio, or retention indices, further support diagnosis and, in standardized protocols, provide an approximate assessment of amyloid burden with potential prognostic value. Collectively, these features position PYP scanning, particularly when combined with SPECT/CT imaging, as a high-specificity diagnostic tool that improves diagnostic accuracy and reduces false-positive interpretations related to blood pooling, often obviating the need for endomyocardial biopsy in clinically compatible cases without evidence of light-chain amyloidosis, thereby streamlining diagnosis and facilitating earlier initiation of disease-modifying therapy.

In our patients, management was complicated by age, comorbidities, and treatment intolerance. One patient experienced gastrointestinal side effects from acoramidis, necessitating a switch to vutrisiran. Because gastrointestinal intolerance is a recognized limitation of acoramidis, we maintained close clinical surveillance from the outset and were able to modify therapy promptly, avoiding significant delay. Heart failure therapy was carefully adjusted, including discontinuation of beta-blockers in restrictive physiology, continuation of RAAS inhibition and SGLT2 inhibitors when tolerated, and anticoagulation in atrial fibrillation, recognizing that anticoagulation is generally recommended in cardiac amyloidosis irrespective of the CHA₂DS₂-VASc score, given the increased thromboembolic risk. One patient (Case 3) succumbed to pneumonia in the setting of complications related to his comorbid conditions, whereas the remaining patients demonstrated improvement in both clinical symptoms and cardiac biomarkers.

## Conclusions

Cardiac amyloidosis is no longer a cardiac disease that can only be diagnosed at tertiary centers. This report highlights that awareness of the multisystem presentation of cardiac amyloidosis among rural cardiologists may help reduce missed or delayed diagnosis. Recognizing key red‑flag features, such as disproportionately elevated NT-proBNP, persistently positive troponin levels, low-voltage or pseudoinfarct patterns on ECG, unexplained left ventricular hypertrophy, and associated extracardiac manifestations, such as peripheral neuropathy, should prompt timely evaluation. By leveraging accessible tools, such as echocardiography, serum and urine monoclonal protein screening, and nuclear scintigraphy, when available, clinicians can implement a practical diagnostic pathway within community settings. Early recognition and implementation of disease-modifying therapy, coupled with supportive heart failure management, may improve quality of life and clinical outcomes, even in older and medically complex patients.

## References

[1] Gertz MA, Dispenzieri A (2020). Systemic amyloidosis recognition, prognosis, and therapy. A systematic review. JAMA.

[2] Garcia-Pavia P, Rapezzi C, Adler Y (2021). Diagnosis and treatment of cardiac amyloidosis: a position statement of the ESC Working Group on Myocardial and Pericardial Diseases. Eur Heart J.

[3] Vergaro G, Aimo A, Barison A, Genovesi D, Buda G, Passino C, Emdin M (2020). Keys to early diagnosis of cardiac amyloidosis: red flags from clinical, laboratory and imaging findings. Eur J Prev Cardiol.

[4] Cotella J, Randazzo M, Maurer MS (2024). Limitations of apical sparing pattern in cardiac amyloidosis: a multicentre echocardiographic study. Eur Heart J Cardiovasc Imaging.

[5] Ladefoged B, Dybro A, Povlsen JA, Vase H, Clemmensen TS, Poulsen SH (2020). Diagnostic delay in wild type transthyretin cardiac amyloidosis - a clinical challenge. Int J Cardiol.

[6] Magdi M, Mostafa MR, Abusnina W (2022). A systematic review and meta-analysis of the prevalence of transthyretin amyloidosis in heart failure with preserved ejection fraction. Am J Cardiovasc Dis.

[7] (2025). AHA. Classes and stages of heart failure. https://www.heart.org/en/health-topics/heart-failure/what-is-heart-failure/classes-of-heart-failure.

[8] Grogan M, Scott CG, Kyle RA (2016). Natural history of wild-type transthyretin cardiac amyloidosis and risk stratification using a novel staging system. J Am Coll Cardiol.

[9] Dimza M, Vasilakis G, Grodin JL (2025). Transthyretin amyloid cardiomyopathy: the plot thickens as novel therapies emerge. US Cardiol.

[10] Maurer MS, Schwartz JH, Gundapaneni B (2018). Tafamidis treatment for patients with transthyretin amyloid cardiomyopathy. N Engl J Med.

[11] Gillmore JD, Judge DP, Cappelli F (2024). Efficacy and safety of acoramidis in transthyretin amyloid cardiomyopathy. N Engl J Med.

[12] Fontana M, Berk JL, Gillmore JD (2025). Vutrisiran in patients with transthyretin amyloidosis with cardiomyopathy. N Engl J Med.

[13] Sheikh A, Miao ZM, Claggett B (2025). Efficacy and safety of vutrisiran in transthyretin amyloid cardiomyopathy across the age spectrum: the HELIOS-B trial. Eur J Heart Fail.

